# Chemistry of Atmospheric Fine Particles During the COVID‐19 Pandemic in a Megacity of Eastern China

**DOI:** 10.1029/2020GL091611

**Published:** 2021-01-18

**Authors:** Lei Liu, Jian Zhang, Rongguang Du, Xiaomi Teng, Rui Hu, Qi Yuan, Shanshan Tang, Chuanhua Ren, Xin Huang, Liang Xu, Yinxiao Zhang, Xiaoye Zhang, Congbo Song, Bowen Liu, Gongda Lu, Zongbo Shi, Weijun Li

**Affiliations:** ^1^ Key Laboratory of Geoscience Big Data and Deep Resource of Zhejiang Province Department of Atmospheric Sciences School of Earth Sciences, Zhejiang University Hangzhou China; ^2^ Hangzhou Meteorological Bureau Hangzhou China; ^3^ School of Environment Hangzhou Institute for Advanced Study University of Chinese Academy of Sciences Hangzhou China; ^4^ School of Atmospheric Sciences Nanjing University Nanjing China; ^5^ State Key Laboratory of Severe Weather/Key Laboratory of Atmospheric Chemistry of China Meteorological Administration Chinese Academy of Meteorological Sciences Beijing China; ^6^ School of Geography, Earth and Environmental Sciences University of Birmingham Birmingham UK; ^7^ Department of Economics University of Birmingham Birmingham UK

**Keywords:** air pollution, chemical composition, COVID‐19, fine particles, megacity

## Abstract

Air pollution in megacities represents one of the greatest environmental challenges. Our observed results show that the dramatic NO_x_ decrease (77%) led to significant O_3_ increases (a factor of 2) during the COVID‐19 lockdown in megacity Hangzhou, China. Model simulations further demonstrate large increases of daytime OH and HO_2_ radicals and nighttime NO_3_ radical, which can promote the gas‐phase reaction and nocturnal multiphase chemistry. Therefore, enhanced NO_3_^−^ and SO_4_^2−^ formation was observed during the COVID‐19 lockdown because of the enhanced oxidizing capacity. The PM_2.5_ decrease was only partially offset by enhanced aerosol formation with its reduction reaching 50%. In particular, NO_3_^−^ decreased largely by 68%. PM_2.5_ chemical analysis reveals that vehicular emissions mainly contributed to PM_2.5_ under normal conditions in Hangzhou. Whereas, stationary sources dominated the residual PM_2.5_ during the COVID‐19 lockdown. This study provides evidence that large reductions in vehicular emissions can effectively mitigate air pollution in megacities.

## Key Points

- Air pollutants PM_10_, PM_2.5_, NO_x_, CO, and SO_2_ decreased whereas O_3_ increased during the COVID‐19 lockdown in megacity Hangzhou
- Enhanced NO_3_^−^ and SO_4_^2−^ formation caused by the enhanced oxidizing capacity partially offset the decrease of PM_2.5_ in megacity Hangzhou
- The contribution of vehicular emissions to PM_2.5_ was over stationary sources under normal conditions in megacity Hangzhou

## 1 Introduction

Atmospheric particulate matter (PM) exerts significant impacts on air quality, climate change, and public health (IPCC, 2013; West et al., 2016). The megacities in developing countries, such as China and India, are facing severe air pollution, especially the fine particle (PM_2.5_) problem because of their fast‐growing economy and urbanization in past decades (Fu & Chen, 2017; Gurjar et al., 2016). To mitigate air pollution, the Chinese State Council implemented the “Air Pollution Prevention and Control Action Plan” in 2013 (Chinese State Council, 2013). As a consequence, anthropogenic emissions of major air pollutants decreased largely (e.g., 59% for SO_2_, 21% for NO_x_, and 33% for primary PM_2.5_) in China during 2013–2017 (Q. Zhang & Geng, 2019). However, despite large reductions in primary emissions, heavy haze episodes still occur in China under unfavorable meteorological conditions and regional pollutant transport. Questions are raised about to what extent the emission reduction achieved can avoid the occurrence of haze episodes.

Many strict short‐term emission controls have been taken by the Chinese government and succeeded in several important national activities, such as the 2008 Beijing Olympic Games, 2014 Asia‐Pacific Economic Cooperation (APEC) summit, 2015 China Victory‐Day Parade, and 2016 G20 summit. These activities are valuable to the studies on the impacts of emission controls on air quality and to understand the roles of emissions and aerosol chemistry in haze formation (H. Li et al., 2016; Sun et al., 2016). For example, during the Beijing Olympic Games, strict controls were implemented in Beijing to reduce emissions from road traffic, industry, and construction sites (T. Wang et al., 2010). The primary gaseous pollutants and fine particles from vehicle emissions and coal combustion decreased significantly, whereas secondary components increased during the first two weeks (T. Wang et al., 2010). This was mainly attributed to the regional transport of secondary aerosols formed outside of Beijing where no emission controls were implemented (Sun et al., 2016). Far stricter emission controls were taken in Beijing and surrounding regions to reduce anthropogenic emissions during the 2014 APEC summit. The daily average PM_2.5_ concentration was reduced to 47.5 μg m^−3^ with reductions of 51%–57% for secondary inorganic aerosols and 37% for secondary organic aerosols, however, regional transport still contributed 44%–57% of the total PM mass (Lin et al., 2017; Sun et al., 2016). Because these controls only concentrated in small regions with short durations, there are great uncertainties in assessing the impacts of emission controls on PM reduction over a small region considering the interference from transboundary air pollutants.

Due to the spread of novel coronavirus disease (COVID‐19) at the end of 2019, the Chinese government implemented strict restrictions on outdoor human activities, including public transport suspended, travel in and out of cities prohibited, schools and entertainment venues closed, and public gatherings banned in late January 2020 (Tian et al., 2020). The nationwide controls provide a unique opportunity to assess the complex response of aerosol chemistry and changes in atmospheric components to the reduction of primary emissions, which further provides an insight into the achievability of air quality improvement in the future. So far, many studies have used satellite data or model simulations to quickly assess the changes in major air pollutants (e.g., NO_2_, O_3_, and PM_2.5_) at large scales in China and around the world after the outbreak of COVID‐19 pandemic (Bauwens et al., 2020; Fan et al., 2020; Huang et al., 2020a; Le et al., 2020; L. Li et al., 2020; Liu et al., 2020; Muhammad et al., 2020; P. Wang et al., 2020; Zhao et al., 2020). However, there is still a lack of detailed chemical composition analysis of PM_2.5_ based on field observations at the city scale, which will be more important for local policymakers.

In this study, meteorological parameters, six criteria air pollutants (PM_10_, PM_2.5_, CO, SO_2_, NO_x_, and O_3_), and chemical components in PM_2.5_ were measured in Hangzhou from 1 January to 31 March 2020. The impacts of lockdown restrictions on the changes in six criteria air pollutants before, during, and after the COVID‐19 lockdown were investigated. Furthermore, chemical components in PM_2.5_ during different stages were characterized in detail to elucidate the changes in aerosol chemistry due to emission reductions. These results can provide regulation strategies for the government to facilitate air quality improvement in the future.

## 2 Methodology

### 2.1 Observation Site

Hangzhou, capital of Zhejiang province, is one of the most developed cities in the south of Yangtze River Delta (YRD) and the host city of the 19th Asian Games in 2022. It has a population of 9.8 million and 2.88 million motor vehicles, and the tertiary industry (e.g., digital economy) is the dominant industry accounting for 63.9% of its Gross Domestic Product according to the Hangzhou Statistical Yearbook 2019. The observation was conducted from 1 January to 31 March 2020 at the Hangzhou National Reference Climatological Station (NRCS, 30°14'N, 120°10'E; 41.7 m above sea level) in the center of Hangzhou (Figure S1). The NRCS, as a typical urban site, is surrounded by residential and commercial buildings, and there are no local industrial sources around the site (Xu et al., 2020).

### 2.2 Instrumentation

#### 2.2.1 Online Measurements

The concentrations of PM_10_ and PM_2.5_ were measured by two particulate matter monitors (Model 5030 SHARP monitor, ThermoFisher Scientific), respectively. The concentrations of NO_x_ (= NO + NO_2_), SO_2_, CO, and O_3_ were detected by a set of commercial gas analyzers (i.e., TEI 42i NO_x_, 43i SO_2_, 48i CO, and 49i O_3_ analyzers, ThermoFisher Scientific). All the instruments are installed on the top floor of the main building in the NRCS. The sampling inlet was mounted 1.5 m above the rooftop. Ambient air was pumped into analyzers through PFA Teflon™ tubes connected to the sampling inlet with a manifold. The meteorological parameters including temperature, relative humidity (RH), atmospheric pressure, wind speed, wind direction, precipitation, and solar radiation were monitored by an automatic weather station. All the online data were hourly averaged and present at local time (Beijing time, UTC+8) in this paper.

#### 2.2.2 Sample Collection and Off‐Line Measurements

Ambient PM_2.5_ samples were collected on 47 mm quartz fiber filters (Whatman) using a sampler (PQ200, BGI) at a flow rate of 16.67 L min^−1^ for 23 h 50 min (i.e., 10:00 a.m. to 9:50 a.m. the next day). Field blank samples were collected for ∼15 min without starting the sampler. The quartz fiber filters were prebaked at 450°C for 6 h to remove any possible contaminants. A total of 60 PM_2.5_ samples were collected. All the samples were sealed in aluminum foil bags and stored in a refrigerator at −20°C until analysis.

Trace metal concentrations (e.g., Al, Ti, V, Cr, Mn, Fe, Ni, Cu, Zn, As, Se, Sr, Ba, and Pb) were acquired with an X‐ray fluorescence spectrometer (Epsilon 4, PANalytical). The concentrations of five cations (Na^+^, NH_4_^+^, K^+^, Mg^2+^, and Ca^2+^) and three anions (Cl^−^, SO_4_^2−^, and NO_3_^−^) were obtained by an ion chromatograph (Dionex ICS 600, ThermoFisher Scientific). Organic carbon (OC) and elemental carbon (EC) were analyzed by an OCEC analyzer (Model 5L, Sunset Laboratory Inc.). The NIOSH870 temperature protocol with thermal‐optical transmittance for charring correction was adopted. Organic matter (OM) concentration was estimated via multiplying OC concentration by a factor of 1.6 based on previous studies (Xing et al., 2013; G. J. Zheng et al., 2015).

### 2.3 Model Simulation

A machine‐learning based random forest algorithm, similar to Grange et al. (2018) and Vu et al. (2019), was applied to decouple the effects of meteorological conditions on the air pollutants acquired by online measurements. The detailed description of this method and its performance on different air pollutants are introduced in the supporting information (Text S1 and Table S1). The differences between the observed and deweathered concentrations of air pollutants can be regarded as meteorology related variations (H. Zheng et al., 2020). The concentration‐weighted trajectory (CWT) model in Igor‐based tool “ZeFir” developed by Petit et al. (2017) was adopted to identify the potential source regions of PM_2.5_ observed at the receptor site in Hangzhou (Text S2, supporting information). Air quality in Hangzhou before and after the COVID lockdown was simulated using the Weather Research and Forecasting model coupled with Chemistry (WRF‐Chem, Text S3, supporting information). The model configurations followed Huang et al. (2020a). The predicted air pollutants and chemical species in PM_2.5_ agree well with observations (Figures S2 and S3).

## 3 Results and Discussion

### 3.1 Overview of Meteorological Conditions and Air Pollutants

Based on the time nodes of notifications and responses on COVID‐19 epidemic prevention and control released by Zhejiang Province (Table S2), the whole observation period is divided into four stages: pre‐COVID (1–23 January), Spring Festival (24 January to 3 February), COVID lockdown (4–19 February), and post‐COVID (20 February to 31 March). Since the Chinese government imposed the strictest controls in Wuhan on 23 January 2020, two days before the Chinese New Year, the first‐level emergency response was immediately imposed by Zhejiang province. On 4 February, the lockdown measures were issued by the Hangzhou government (Yuan et al., 2020). The Spring Festival and COVID lockdown were covered by the COVID‐19 pandemic period when similar strictest controls were imposed by local governments. The Spring Festival stage was separated to eliminate the influence of fireworks during the Spring Festival holiday. During the post‐COVID stage, the spread of COVID‐19 was under control and normal living and production activities were restored gradually.

**Figure 1:**
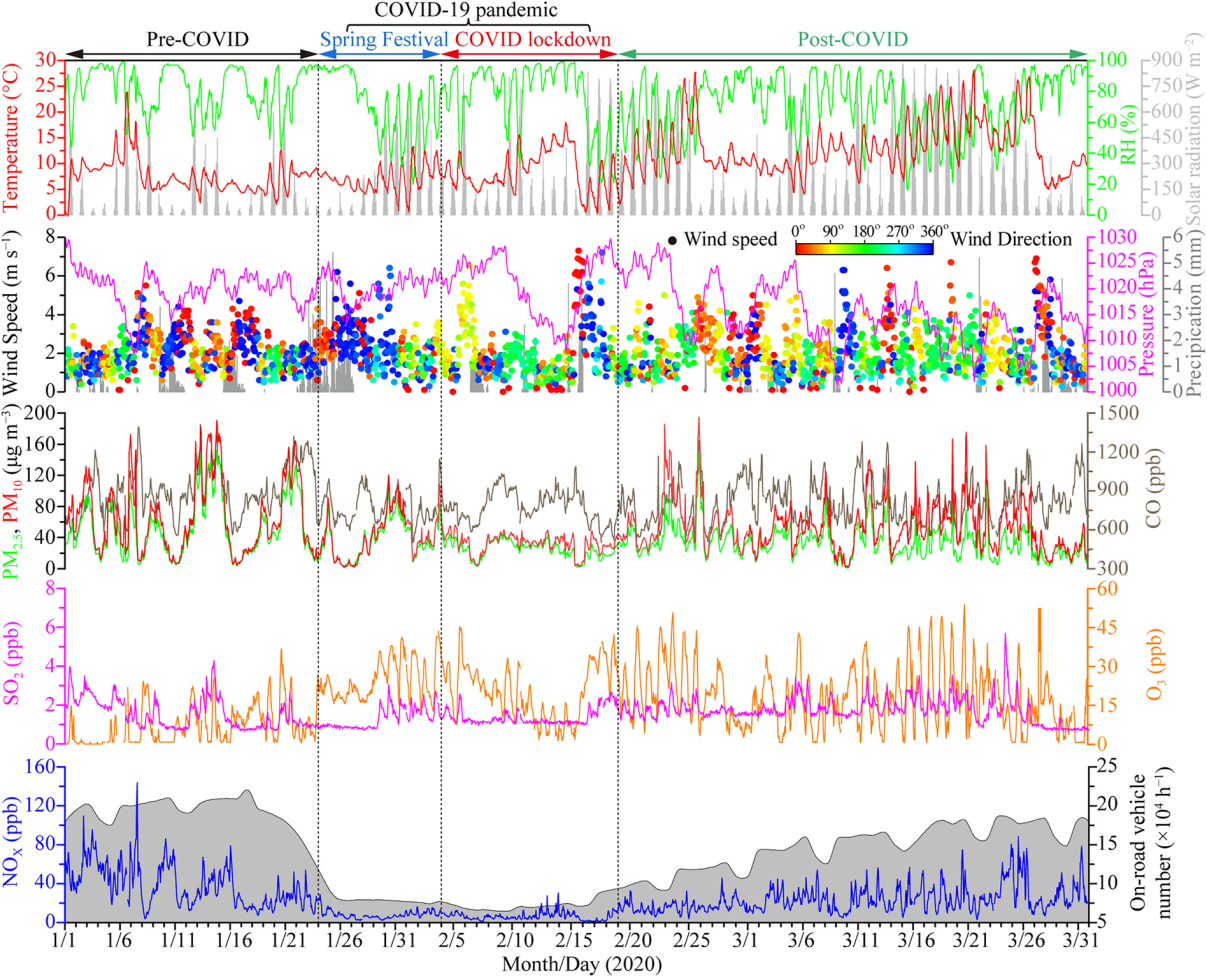
Time series of hourly average meteorological parameters, particulate matter, gaseous pollutants, and daily average on‐road vehicle number per hour in urban Hangzhou during the observation period. The traffic data were obtained from Hangzhou Traffic Police Division. The four defined stages are marked on the top of this figure.

Figure 1 shows the time series of hourly average meteorological parameters, particulate matter (PM_10_ and PM_2.5_), gaseous pollutants (CO, SO_2_, O_3_, and NO_x_), and daily average traffic volume during the entire observation. The on‐road vehicles decreased dramatically by 84% during the COVID lockdown compared with those during the pre‐COVID stage and increased gradually during the post‐COVID stage. Although the wind direction was dominated by northerlies during the pre‐COVID stage and by southerlies during the last three stages, on the whole, similar average meteorological parameters in terms of temperature (i.e., 7.5, 6.9, 8.4, and 13.0°C), RH (i.e., 82%, 72%, 76%, and 72%), and wind speed (i.e., 2.0, 2.3, 2.0 and 2.1 m s^−1^) were present among the pre‐COVID, Spring Festival, COVID lockdown, and post‐COVID stages (Table S3).

The observed and deweathered average concentrations of PM_10_, PM_2.5_, CO, SO_2_, NO_x_, and maximum daily 8‐h average O_3_ (MDA8 O_3_) during four stages are listed in Tables S3 and S4, respectively. We found that emission reductions dominated the changes of air pollutants with limited influence from the meteorology during the Spring Festival and COVID lockdown (Figure S4). Furthermore, the CWT analysis clearly shows that potential sources of PM_2.5_ during the pre‐COVID and Spring Festival stages were mainly originated from the regional (e.g., Jiangsu and Anhui) and local (i.e., northern Zhejiang) contributions in the YRD (Figures S5a and S5b); during the COVID lockdown and post‐COVID stages, the local area (i.e., northern Zhejiang) was the main potential sources of PM_2.5_ (Figures S5c and S5d). Overall, the potential sources of PM_2.5_ in Hangzhou during the four stages were mainly concentrated in the YRD and less affected by long‐range transport from other regions (e.g., North China Plain).

**Figure 2:**
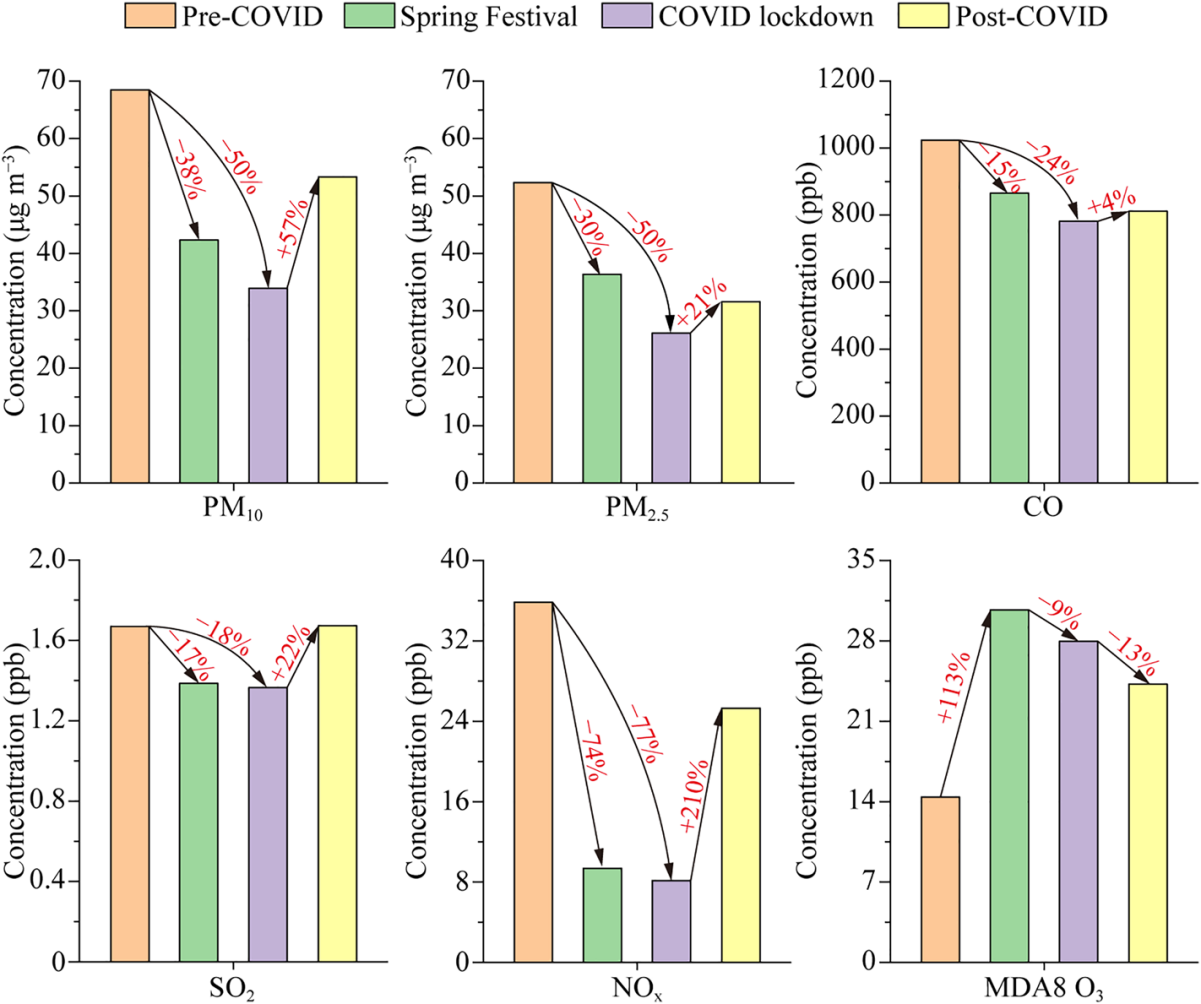
Changes in average deweathered concentrations of particulate matter and gaseous pollutants among four stages.

Figure 2 shows that after decoupling the effects of meteorology, all the air pollutants (except O_3_) displayed the lowest concentrations (34.2 μg m^−3^ for PM_10_, 26.3 μg m^−3^ for PM_2.5_, 782 ppb for CO, 1.4 ppb for SO_2_, and 8.1 ppb for NO_x_) during the COVID lockdown. On the contrary, MDA8 O_3_ concentration increased significantly from 14.4 ppb during the pre‐COVID stage to 30.7 ppb during the Spring Festival stage, and then slightly decreased to 27.9 ppb during the COVID lockdown. Compared with those during the pre‐COVID stage, NO_x_ had the largest reduction of 77%, followed by PM_10_ 50%, PM_2.5_ 50%, CO 24%, and SO_2_ 18% during the COVID lockdown. The variation trend of NO_x_ is consistent with the traffic volume (Figure 1), which indicates that the significant reduction of NO_x_ was linked to the sharp decrease of traffic volume in the city. Deweathered SO_2_ only reduced by 18%, significantly less than that of NO_x_ (77%). This result suggests that COVID restrictions had a much more important influence on vehicular emissions but not on the large power plants and heavy industries (e.g., steel and petrochemical industries), considering there is no domestic heating with coal in southern China and the economy of Hangzhou is dominated by the third industry. Deweathered MDA8 O_3_ increased significantly by a factor of 2 during the COVID‐19 pandemic period compared with that during the pre‐COVID stage. The increase of O_3_ may result from the sharp decrease of NO emitted from the traffic, which weakened the NO‐titration effect on O_3_ (Wu et al., 2019). Besides, the increasing solar radiation during the Spring Festival and COVID lockdown may lead to positive changes in the net photochemical formation rate of O_3_ (Figure S6), which also contributed to the increase of O_3_ (K. Li et al., 2019). When the restrictions were loosened progressively during the post‐COVID stage, all the pollutants rebounded except for O_3_. The solar radiation was the highest during the post‐COVID stage but O_3_ still decreased (Figure S6), which indicates that the sharp NO decrease was the major factor contributing to the significant increase of O_3_ during the COVID‐19 pandemic period.

### 3.2 Chemical Composition of PM_2.5_

**Figure 3:**
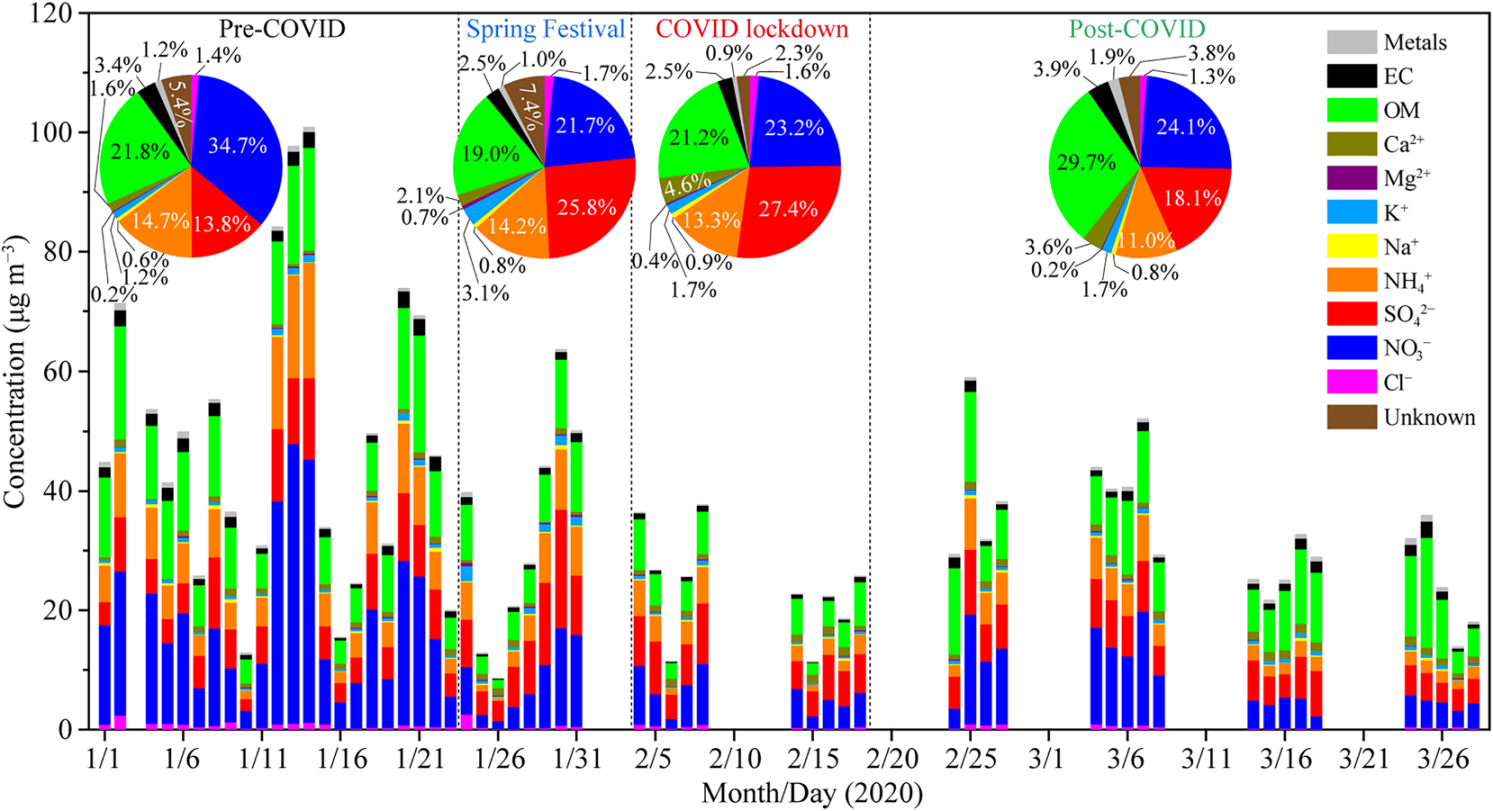
Daily concentrations of chemical species in PM_2.5_ during the entire observation period. Pie charts (inset) present average mass fractions of chemical species in PM_2.5_ during four stages.

**Figure 4:**
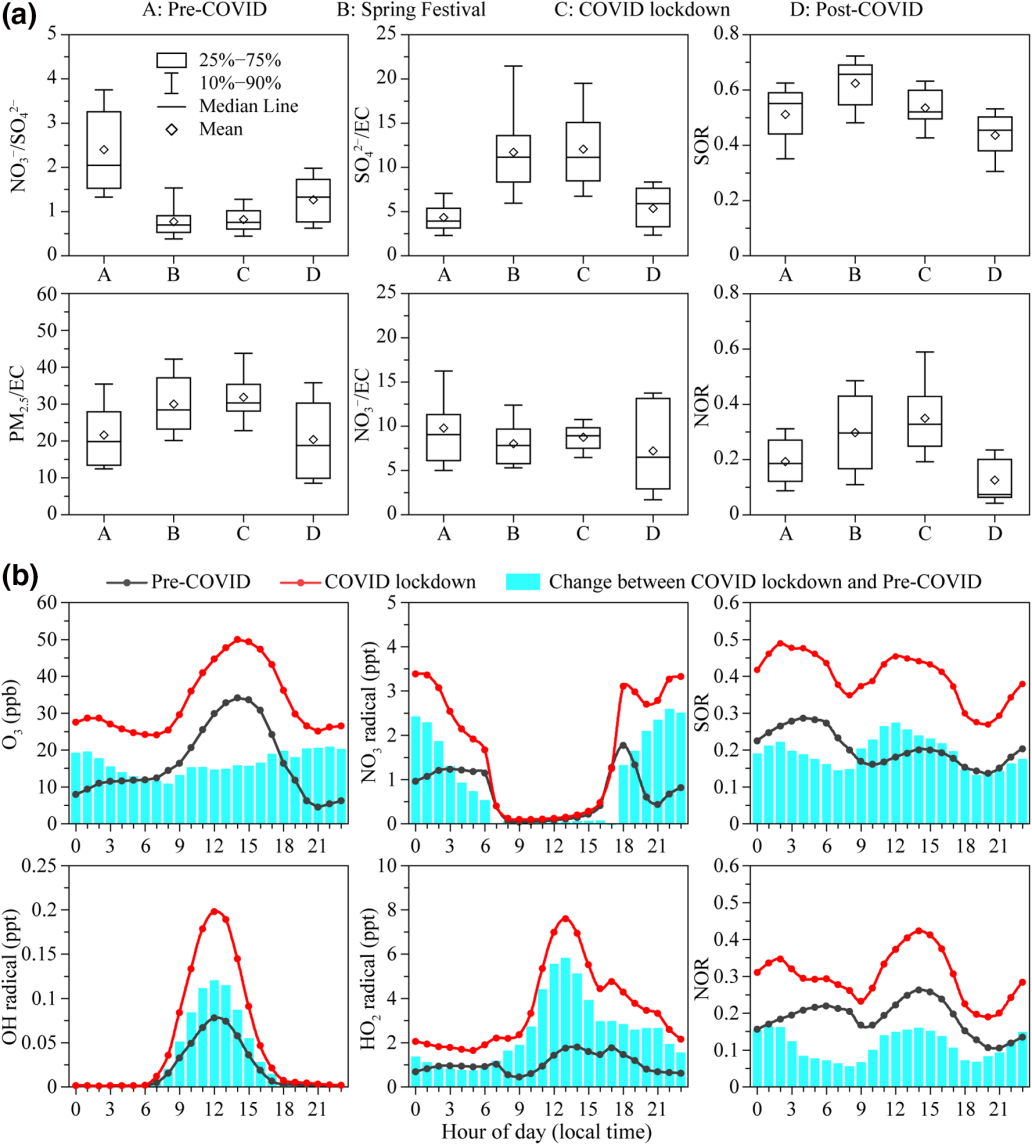
(a) Variations in average NO_3_^−^/SO_4_^2−^, PM_2.5_/EC, SO_4_^2−^/EC, NO_3_^−^/EC, SOR, and NOR during the four stages; (b) diurnal cycles of simulated O_3_, NO_3_ radical, OH radical, HO_2_ radical, SOR, and NOR during the preCOVID and COVID lockdown stages.

The daily concentrations of chemical species and their mass fractions in PM_2.5_ were analyzed to investigate their changes among four stages (Figure 3). During the pre‐COVID stage, the average concentrations of NH_4_^+^, NO_3_^−^, SO_4_^2−^, OM, and EC were 7.6, 17.8, 7.1, 11.2, and 1.8 μg m^−3^, respectively, with NO_3_^−^ being the dominant component (34.7%), followed by OM (21.8%), NH_4_^+^ (14.7%), SO_4_^2−^ (13.8%), and EC (3.4%) (Figure 3 and Table S5). During the Spring Festival and COVID lockdown, NO_3_^−^ concentration decreased dramatically to 7.8 and 5.7 μg m^−3^, respectively, with the corresponding reductions of 56% and 68% compared with that during the pre‐COVID stage (Figure S7 and Table S5). It is clear that the NO_3_^−^ change is strongly associated with the sharp decline of its precursor NO_x_ as shown in Figure 2. Compared with that during the pre‐COVID stage, SO_4_^2−^ concentration increased slightly from 7.1 to 9.3 μg m^−3^ during the Spring Festival and then decreased to 6.7 μg m^−3^ during the COVID lockdown (Figure S7 and Table S5). SO_4_^2−^ became the dominant component (25.8% and 27.4%), exceeding NO_3_^−^ (21.7% and 23.2%) during the Spring Festival and COVID lockdown (Figure 3). The NO_3_^−^/SO_4_^2−^ ratio can indicate the relative importance of vehicular versus stationary sources (H. Li et al., 2016). A higher NO_3_^−^/SO_4_^2−^ ratio (>2) was observed during the pre‐COVID stage compared with those (<1) during the Spring Festival and COVID lockdown (Figure 4a). This result reveals the predominance of vehicular sources over stationary sources during the pre‐COVID stage; conversely, stationary sources dominated during the COVID‐19 pandemic period. The result is reasonable because the large heavy industries, coal‐fired power plants, and household cooking were still running, although some small private industries were closed in China during the COVID‐19 pandemic period. The relatively small change of SO_2_ further supports this argument (Figure 2).

Mass concentrations of OM and NH_4_^+^ largely decreased by 39% and 32% during the Spring Festival and 54% and 57% during the COVID lockdown, respectively, compared with those during the pre‐COVID stage (Figure S7). However, the contributions of OM (19.0% and 21.2%) and NH_4_^+^ (14.2% and 13.3%) during the Spring Festival and COVID lockdown only showed a slight decrease compared with those (OM 21.8% and NH_4_^+^ 14.7%) during the pre‐COVID stage (Figure 3). EC had the lowest contributions (2.5%) during the Spring Festival and COVID lockdown with EC mass concentrations decreasing respectively by 48% and 63% compared with that during the pre‐COVID stage (Figures 3 and S7). Mass concentrations of trace metals showed a decrease of 15%–86% during the COVID lockdown compared with those during the pre‐COVID stage (Figure S8 and Table S6). In general, the primary inorganic aerosols in PM_2.5_ (i.e., EC, Cl^−^, K^+^, Ca^2+^, Mg^2+^, Na^+^, and trace metals) decreased by 38% and secondary inorganic aerosols (i.e., NO_3_^−^, SO_4_^2−^, and NH_4_^+^) decreased by 52% during the COVID lockdown compared with those during the pre‐COVID stage. The restrictions on anthropogenic activities, in particular the sharp decline of on‐road vehicles induced a significant NO_3_^−^ decrease (68%). Whereas, the natural dust was not affected by the COVID lockdown although halted construction activities can reduce the anthropogenic dust. Besides, the primary emissions from the heavy industries were less affected. Therefore, the quicker decrease of secondary inorganic aerosols (52%) than primary inorganic aerosols (38%) was observed during the COVID lockdown.

It is worth noting that the concentrations of K^+^ (1.1 μg m^−3^), Mg^2+^ (0.24 μg m^−3^), Al (131 ng m^−3^), Ti (20 ng m^−3^), Ba (65 ng m^−3^), Sr (17 ng m^−3^), and Cu (14 ng m^−3^) were the highest during the Spring Festival (Figures S7 and S8), indicating a large contribution of fireworks to PM_2.5_ (W. Li et al., 2013). According to China's environmental policy, fireworks are strictly prohibited in the central urban area during the Spring Festival but not in rural areas. Indeed, fireworks were often displayed in rural areas during the Chinese New Year holiday from 24 January to 3 February 2020.

During the post‐COVID stage, OM (29.7% in PM_2.5_) and NO_3_^−^ (24.1% in PM_2.5_) became the dominant components responsible for the rebound of PM_2.5_ with a growth rate of 95% and 45% compared with those during the COVID lockdown (Figures 3 and S7). Meanwhile, mass concentrations of SO_4_^2−^ and NH_4_^+^ did not change largely (Figure S7). As a result, the contributions of SO_4_^2−^ and NH_4_^+^ decreased from 27.4% and 13.3% during the COVID lockdown to 18.1% and 11.0% during the post‐COVID stage, respectively (Figure 3).

### 3.3 Formation of Secondary Aerosols

Since EC only comes from primary combustion emissions and is inert to chemical reactions, the ratios of PM_2.5_, SO_4_^2−^, and NO_3_^−^ to EC can somewhat reflect the relative changes between secondary production and primary emission (J. Zhang et al., 2017; G. J. Zheng et al., 2015). The sulfur oxidation ratio (SOR, molar ratio of SO_4_^2−^ to sum of SO_4_^2−^ and SO_2_) and nitrogen oxidation ratio (NOR, molar ratio of NO_3_^−^ to sum of NO_3_^−^ and NO_2_) has been widely used to indicate the production rates of SO_4_^2−^ and NO_3_^−^ from their corresponding precursors SO_2_ and NO_x_ (Sicard et al., 2020; Yuan et al., 2015).

An increase of PM_2.5_/EC occurred during the Spring Festival and COVID lockdown, followed by a decrease during the post‐COVID stage (Figure 4a). This result indicates that secondary aerosol production was enhanced relative to primary emissions during the Spring Festival and COVID lockdown in comparison with those during the pre‐ and post‐COVID stages. The SO_4_^2−^/EC ratios during the Spring Festival and COVID lockdown were much higher than those during the pre‐ and post‐COVID stages. Meanwhile, the SOR shows a similar variation trend as the SO_4_^2−^/EC (Figure 4a). On the contrary, higher NOR but lower NO_3_^−^/EC ratios were observed during the Spring Festival and COVID lockdown compared with those during the pre‐COVID stage (Figure 4a). The higher SOR and NOR indicate higher production rates of SO_4_^2−^ and NO_3_^−^ from SO_2_ and NO_x_ during the Spring Festival and COVID lockdown. Whereas, the enhanced production rate of NO_3_^−^ did not cause the increase of NO_3_^−^/EC (Figure 4a), which was attributed to the much more significant reduction of NO_x_ (74% and 77%) than EC (48% and 63%) during Spring Festival and COVID lockdown (Figures 2 and S7). Therefore, the NO_3_^−^EC still decreased although the production rate of NO_3_^−^ was enhanced.

In order to have a deep insight into the changes of secondary aerosols responding to the primary emission reductions, we further performed the WRF‐Chem model simulation. Figure S9 shows the predicted average concentrations of SO_4_^2−^, NO_3_^−^ NH_4_^+^, OM, and EC during the pre‐COVID and COVID lockdown stages and their changes between the two stages. The relative changes from the model simulation (SO_4_^2−^ +25.8%, NO_3_^−^ −43.3%, NH_4_^+^ −30.2%, OM −32.5%, and EC −63.9%) are in good agreement with the observations except for SO_4_^2−^ (Figure S9). Figure 4b shows the diurnal cycles of predicted oxidants (i.e., O_3_ and OH, HO_2_, and NO_3_ radicals), SOR, and NOR during the pre‐COVID and COVID lockdown stages. The model simulation reproduced the observed enhancement of O_3_ during the COVID lockdown. The changes in O_3_ concentration between the COVID lockdown and pre‐COVID stage were significant, especially during the nighttime due to the weakened NO‐titration effect. The concentrations of nighttime NO_3_ radical and daytime HO_x_ (i.e., OH and HO_2_) radical were much higher during the COVID lockdown compared with those during the pre‐COVID stage. These results indicate the oxidizing capacity was significantly enhanced during the COVID lockdown. The predicted SOR and NOR during the COVID lockdown were higher than those during the pre‐COVID stage, which consists well with the observed results indicating the enhanced production rates of SO_4_^2−^ and NO_3_^−^. In particular, the increases of SOR and NOR during the COVID lockdown were more obvious at noon (11:00 to 15:00) and midnight (23:00 to 3:00). The two periods coincide with the increases of daytime HO_x_ radical and nighttime O_3_ and NO_3_ radical. We conclude that the increased HO_x_ radical concentration enhanced the gas‐phase reactions of SO_2_ and NO_x_ during the daytime, and the nocturnal multiphase chemistry was promoted due to the increased O_3_ and NO_3_ radical concentrations.

It should be noted that the enhanced secondary aerosol formation during the COVID lockdown partially offset the benefit of primary reductions on the decrease of PM_2.5_ in Hangzhou, causing the decreases of SO_4_^2−^ and NO_3_^−^ less than their precursors (SO_4_^2−^ 6% vs. SO_2_ 18%, NO_3_^−^ 68% vs. NO_x_ 77%). Nevertheless, the NO_3_^−^ concentration still presented a significant decrease due to the dramatic decline of NO_x_, and the lowest PM_2.5_ concentration was observed during the COVID lockdown in Hangzhou (Figures 2 and S7), which differs from the increase of PM_2.5_ in northern China reported by Huang et al. (2020a).

## 4 Conclusions and Implications

The outbreak of COVID‐19 caused large changes in anthropogenic emissions worldwide. Our results demonstrate that reductions in vehicular emissions were more responsible for the PM_2.5_ decline compared with stationary emissions during the COVID lockdown in Hangzhou. Although the dramatic decrease of NO_x_ and other air pollutant emissions led to the significant increase of O_3_, the concentration of O_3_ in Hangzhou still below the ambient air quality standards of China (GB 3095‐2012) and World Health Organization (WHO, 2005). Therefore, the influence of O_3_ increase on the health risk assessment is relatively small. Besides, the increased secondary aerosol production caused by the enhanced oxidizing capacity only partially offset the reduction in PM_2.5_ and PM_2.5_ still decreased largely in Hangzhou. Huang et al. (2020b) reported that 42.4 thousand premature deaths were avoided associated with PM_2.5_ reduction during the lockdown in the YRD. Therefore, stricter standards should be taken on the current vehicular emissions in the YRD. Moreover, new energy (e.g., electricity) vehicles should be further encouraged by the governments to replace fuel vehicles and reduce the emission of NO_x_.

It should be emphasized that the lowest observed average PM_2.5_ concentration was 26.3 μg m^−3^ during the COVID lockdown in Hangzhou, which is still much higher than the WHO's air quality guideline of 10 μg m^−3^ (WHO, 2005), though almost all the human transportations had been restricted. Regional stationary emissions were the major sources contributing to the residual PM_2.5_ during the COVID lockdown in regional air. Therefore, it is more challenging to further reduce PM_2.5_, which needs deeper energy and industrial restructuring and regional joint‐controls in the future. In a word, this study provides evidence that reductions in human activities, especially vehicular emissions can largely mitigate air pollution in megacities.

## Conflict of Interest

The authors declare that they have no conflicting interest.

## Supporting information

Supporting Information S1

## Data Availability

Data sets for this research are available at Figshare (https://doi.org/10.6084/m9.figshare.12919013).
