# Supplementary material for: Chemistry of Atmospheric Fine Particles During the COVID‐19 Pandemic in a Megacity of Eastern China
Source: Geophys Res Lett. 2021 Jan 18;48(2):2020GL091611. doi: 10.1029/2020GL091611 (PMC7883225; doi:10.1029/2020GL091611)
Supplement: Supplementary file 1 — Supporting Information S1 [file GRL-48-2020GL091611-s001.docx]

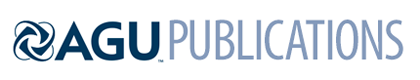


*Geophysical Research Letters*

Supporting Information for

**Chemistry of Atmospheric Fine Particles during the COVID-19 Pandemic**

**in a Megacity of Eastern China**

Lei Liu^1^, Jian Zhang^1^, Rongguang Du^2^, Xiaomi Teng^1^, Rui Hu^2^, Qi Yuan^1^, Shanshan Tang^3^, Chuanhua Ren^4^, Xin Huang^4^, Liang Xu^1^, Yinxiao Zhang^1^, Xiaoye Zhang^5^, Congbo Song^6^, Bowen Liu^7^, Gongda Lu^6^, Zongbo Shi^6^, and Weijun Li^1,^*

^1^Key Laboratory of Geoscience Big Data and Deep Resource of Zhejiang Province, Department of Atmospheric Sciences, School of Earth Sciences, Zhejiang University, Hangzhou 310027, China

^2^Hangzhou Meteorological Bureau, Hangzhou 310051, China

^3^School of Environment, Hangzhou Institute for Advanced Study, University of Chinese Academy of Sciences, Hangzhou 310024, China

^4^School of Atmospheric Sciences, Nanjing University, Nanjing 210023, China;

^5^State Key Laboratory of Severe Weather/Key Laboratory of Atmospheric Chemistry of China Meteorological Administration, Chinese Academy of Meteorological Sciences, Beijing 100081, China

^6^School of Geography, Earth and Environmental Sciences, University of Birmingham, Birmingham B15 2TT, UK

^7^Department of Economics, University of Birmingham, Birmingham, B15 2TT, UK

*Corresponding author: Weijun Li (liweijun@zju.edu.cn)

**Contents of this file**

Text S1 to S3

Figures S1 to S9

Tables S1 to S6

**Text S1. Deweather analysis of air pollutants**

Weather conditions change rapidly, causing variations in the concentration of air pollutants even when the emissions do not change. To decouple the effects of meteorological conditions on the air pollutants, we build random forest (RF) model for each pollutant. 70% of the original data were randomly selected to build the model, which was then evaluated with the rest (30%) of the dataset. Similar to Grange et al. (2018) and Vu et al. (2019), the weather normalization was conducted using the "rmweather" R package, available at: https://cran.r-project.org/web/packages/rmweather/index.html. We only normalized the weather data but not time variables, which is similar to Vu et al. (2019), and resampled from the whole study period, which is similar to Grange et al. (2018). The improved method is more suitable for tracking emission changes. The input features for the model included time variables (i.e., Unix time, Julian day, day of the week, and hour of the day) and meteorological data from surface observations (i.e., temperature, relative humidity, wind speed, wind direction, and pressure). The day of week was categorical variables while all others were numeric. Following Vu et al. (2019), the parameters for the RF models are: a forest of 300 trees (n_tree = 300); the number of variables that may split at each node is 3 (mtry = 3); and the minimum size of terminal nodes is 3 (min_node_size = 3). For every weather normalization, the explanatory variables were resampled from the variables (excluding the time variables) without replacement and randomly allocated to a dependent variable observation. The 1000 predictions were then aggregated using the arithmetic mean and this was the deweathered concentrations.

**Text S2. Concentration-weighted trajectory (CWT) analysis**

The CWT analysis, which couples atmospheric concentrations of pollutants with backward trajectories and uses residence time information, is developed to identify the potential sources of air pollutants observed at the receptor site over large geographical scales (Fleming et al., 2012; Polissar et al., 2001). In this study, 48-h air mass backward trajectories started every one hour arriving at the sampling site (30º14'N, 120º10'E) over 100 m a.g.l. were firstly calculated from the PC-based version of HYSPLIT model in TrajStat software (Stein et al., 2016; Wang et al., 2009) using the meteorological data downloaded from the National Oceanic and Atmospheric Administration (NOAA) website (accessed on: <ftp://arlftp.arlhq.noaa.gov/pub/archives/gdas1>). Then, a user-friendly Igor-based tool “ZeFir” developed by Petit et al. (2017) was adopted for the CWT analysis. The region covered by the trajectories of CWT consists of thousands of cells with a resolution of 0.25°×0.25°. The CWT was simulated according to the following equation:

$\text{C}_{\text{ij}}=\frac{\text{1}}{\sum_{\text{k}\text{=1}}^{\text{N}} \text{τ}_{\text{ijk}}}\sum_{\text{k}\text{=1}}^{\text{N}} \text{C}_{\text{k}}\text{τ}_{\text{ijk}}$ (1)

where *C_ij_* is the average weighted concentration in a grid cell (*i*, *j*); *C_k_* is the measured concentration of pollutant observed on the arrival of trajectory *k*; *τ_ijk_* is the number of trajectory endpoints in grid cell (*i*, *j*) associated with the *C_k_* sample; *N* is the number of samples that have trajectory endpoints in grid cell (*i*, *j*). To further improve the accuracy of CWT analysis, a discrete weighing function in “ZeFir” as shown in equation (2) was applied to downweight cells associated with low residence time.

$\text{W=}\left\{ \begin{aligned} \text{1 }\text{ }\text{ for }\text{ }\log\left( \text{n}\text{+1} \right)\text{ }\text{≥}\text{ }\text{0.85×}\text{max}_{\text{log(}\text{n}\text{+1)}} \\ \text{0.725}\text{ }\text{ for 0.85×}\text{max}_{\text{log(}\text{n}\text{+1)}}\text{ >}\log\left( \text{n}\text{+1} \right)\text{≥}\text{ }\text{0.6}\text{0}\text{×}\text{max}_{\text{log(}\text{n}\text{+1)}} \\ \text{0.475 }\text{ }\text{ for 0.6}\text{0}\text{×}\text{max}_{\text{log(n+1)}}\text{ }\text{>}\log\left( \text{n}\text{+1} \right)\text{≥}\text{ }\text{0.35×}\text{max}_{\text{log(}\text{n}\text{+1)}} \\ \text{0.175 }\text{ }\text{ for }\text{ }\text{ log}\left( \text{n}\text{+1} \right)\text{ }\text{<}\text{ }\text{0.35×}\text{max}_{\text{log(}\text{n}\text{+1)}} \end{aligned} \right.$ (2)

log(*n*+1) represents the density of trajectory.

**Text S3. WRF-Chem model simulation**

To quantitatively understand the response of air quality before and after the COVID-lockdown, regional coupled dynamical and chemical simulations are conducted based on Weather Research and Forecasting model coupled with Chemistry (WRF-Chem) version 3.7.1 (Grell et al., 2005). In this study, the model configurations were equivalent to the study by Huang et al. (2020). The domain with a grid resolution of 20 km covered the eastern China and its surrounding areas to get synoptic forcing. There are 30 vertical layers from the ground level to the top pressure of 50 hPa, in which more than ten layers are settled under 1 km to better describe boundary layer processes. The initial and boundary meteorological conditions were the NCEP global final analysis (FNL) data. NCEP Automated Data Processing (ADP) surface and global upper air observational weather data of wind, temperature and moisture is assimilated to better characterize the regional transport pattern of air pollution.

The base simulation was conducted from 1 January to 15 March, 2020 by using the bottom-up inventory model of Multi-resolution Emission Inventory for China (MEIC), developed by Tsinghua University (Li et al., 2014). Anthropogenic emissions from power plants, residential combustion, industrial processes, on-road mobile sources and agricultural activities were derived from the MEIC database. During the lockdown period, considering the activity level change due to COVID lockdown we then use the up-to-date emission reduction ratio, to quantitatively understand the emission-triggered perturbations in air pollution, which can be found from the study by Huang et al. (2020).


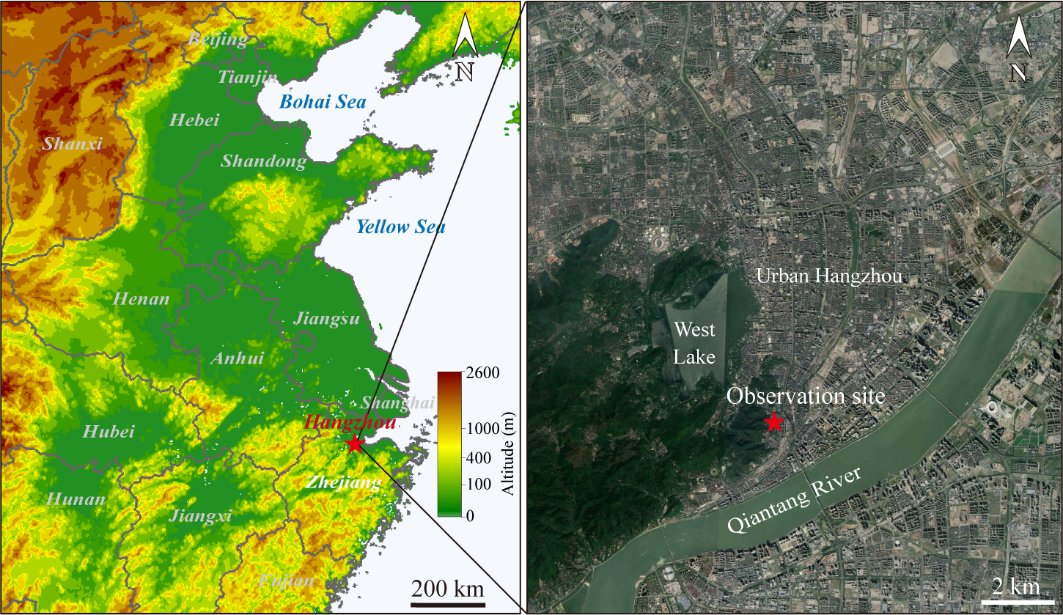


Figure S1. Location of the observation site in Hangzhou city. (Map copyright @2020 Google Maps)


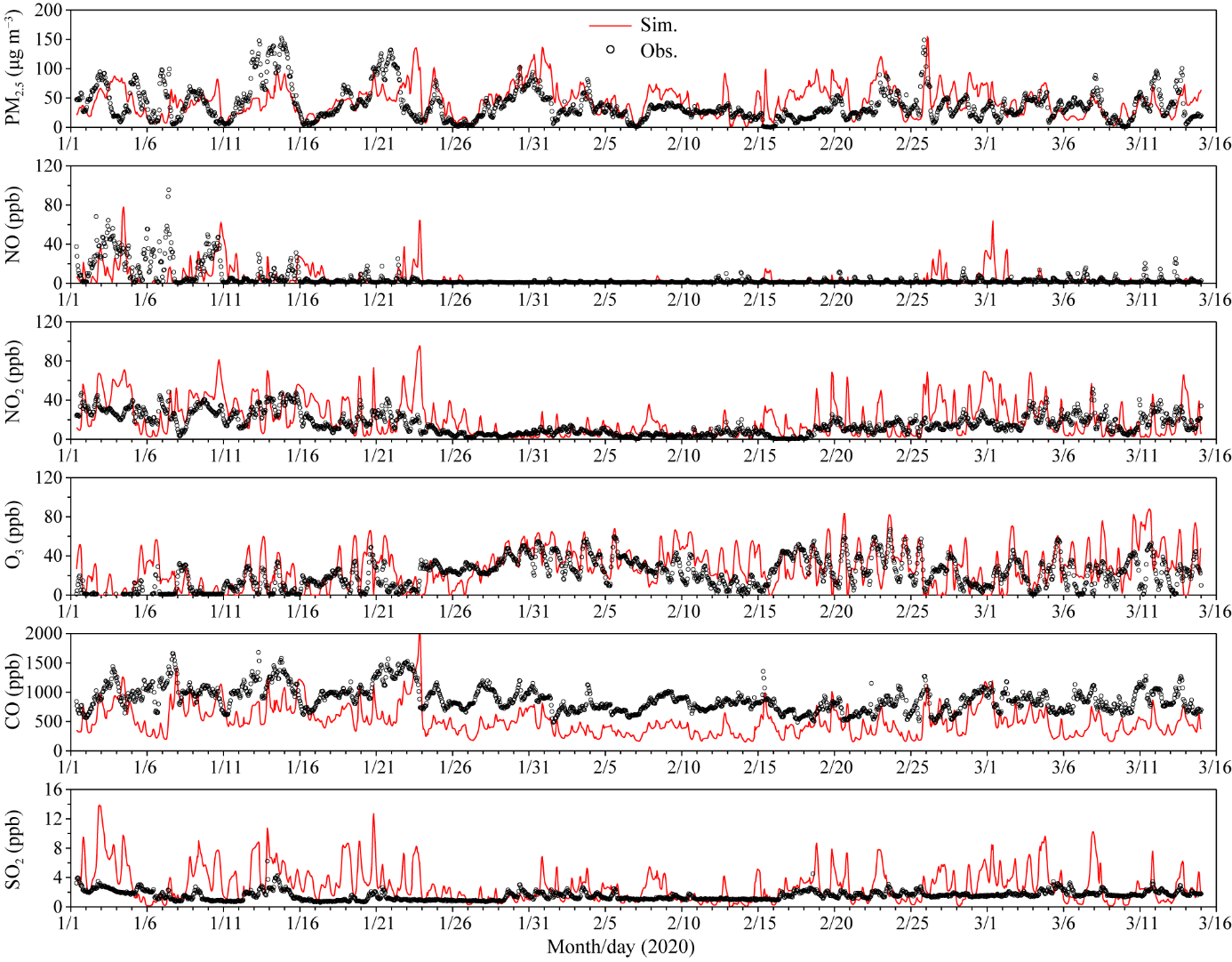


Figure S2. Predicted hourly concentrations (red lines) of PM_2.5_, NO, NO_2_, O_3_, CO, and SO_2_ compared with observations (black circles).


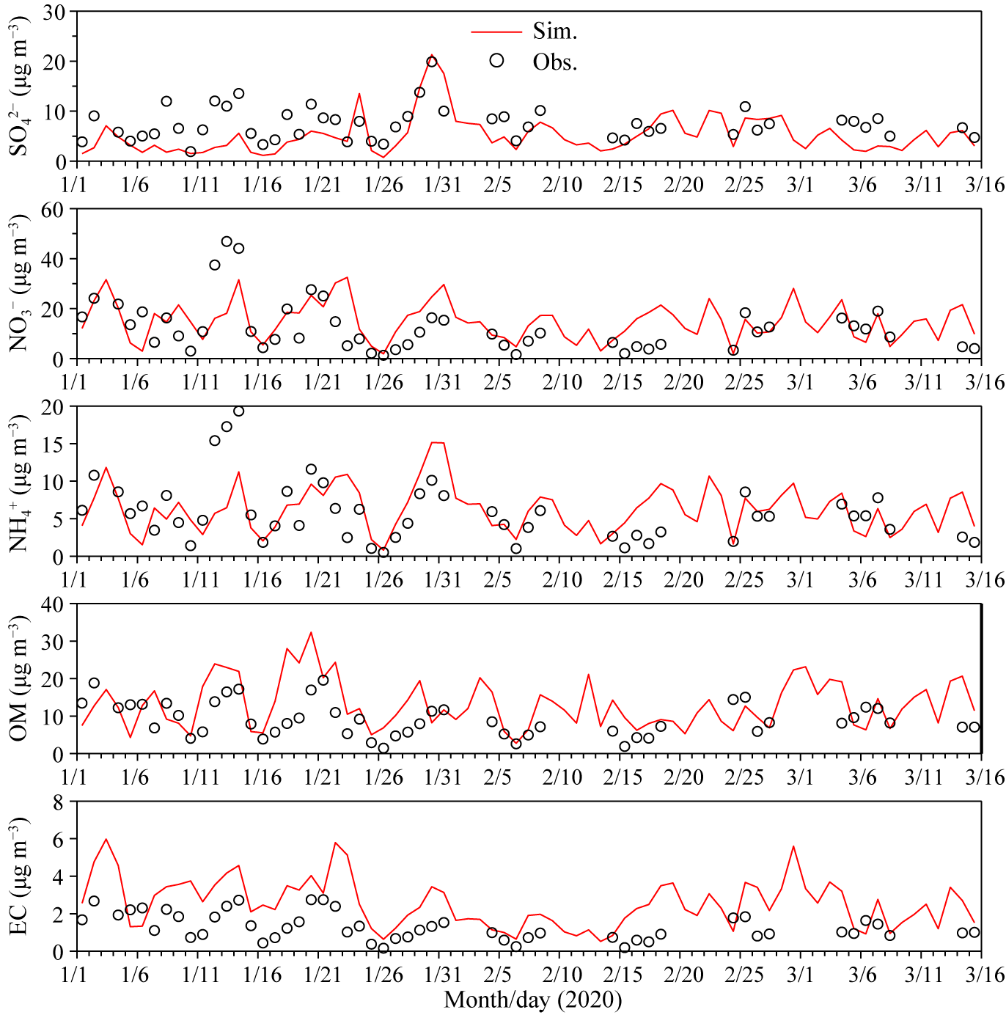


Figure S3. Predicted daily concentrations (red lines) of chemical components (i.e., SO_4_^2−^, NO_3_^−^, NH_4_^+^, OM, and EC) in PM_2.5_ compared with observations (black circles).


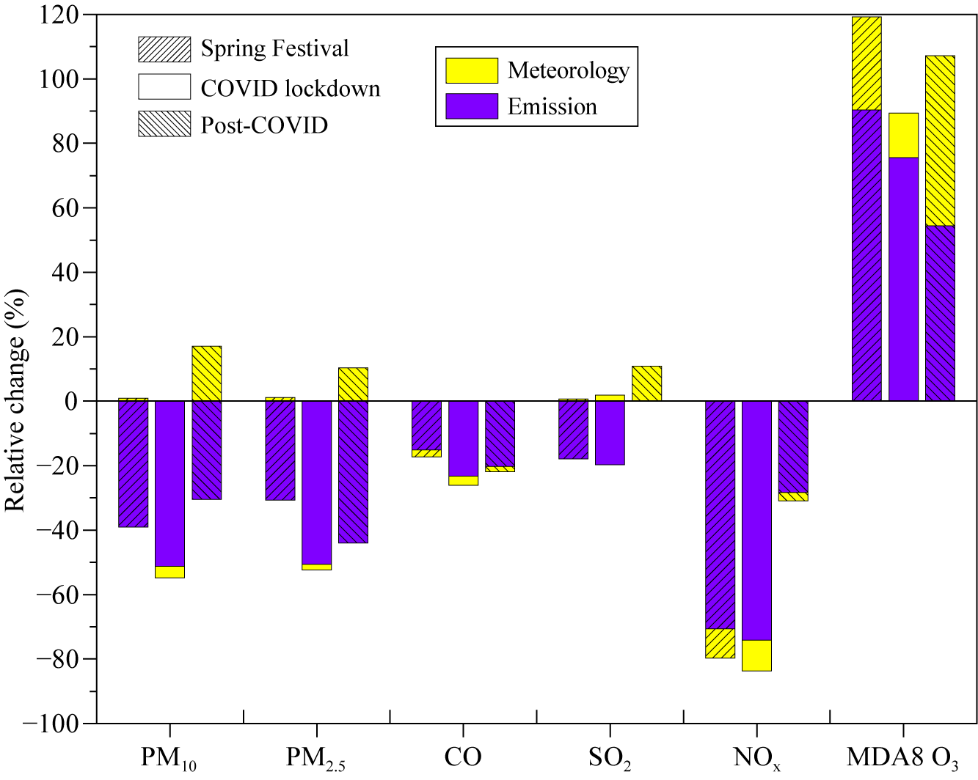


Figure S4. Relative changes of air pollutants during the Spring Festival, COVID lockdown, and post-COVID stages compared with those during the pre-COVID stage caused by meteorology and emission, respectively.


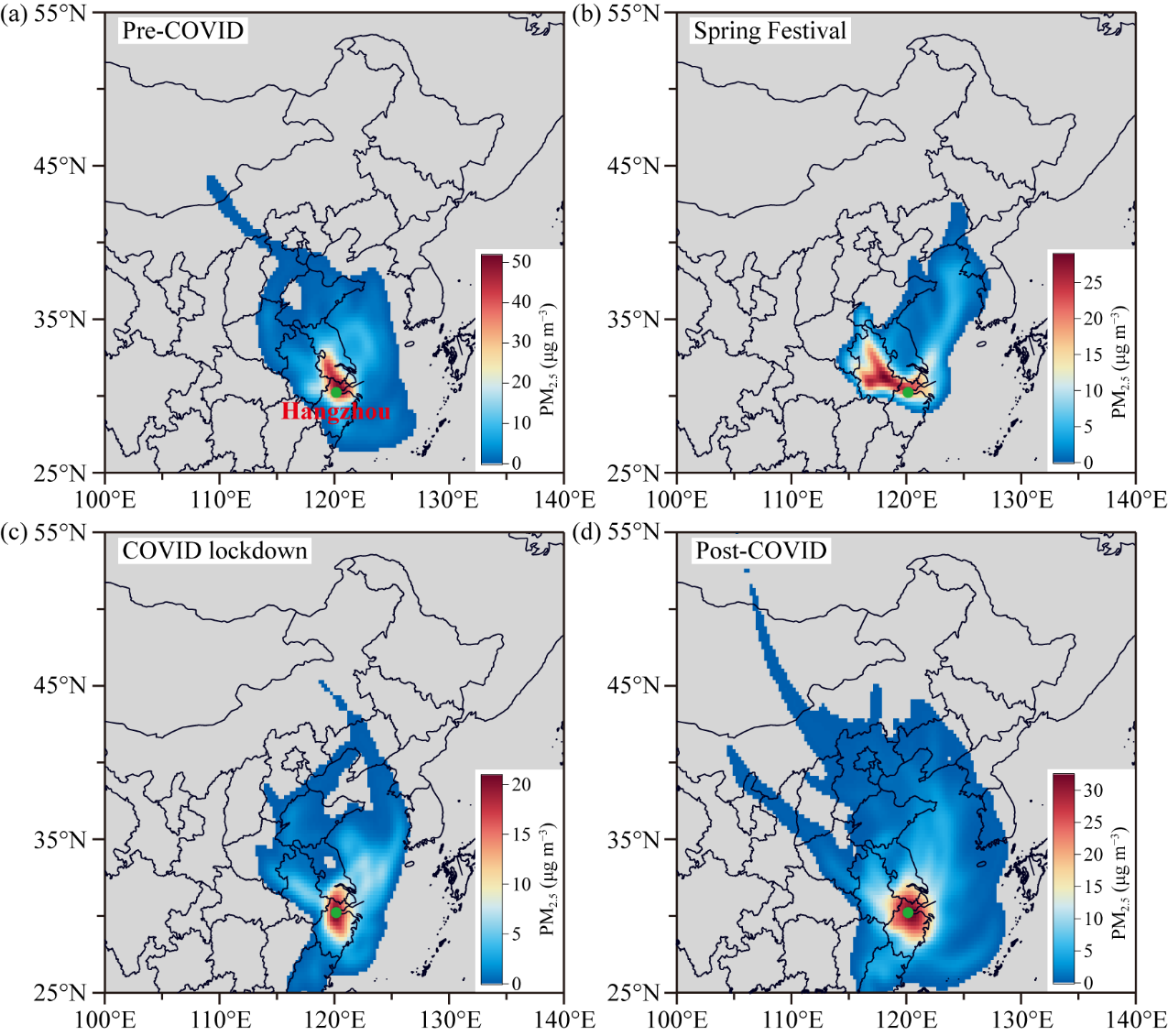


Figure S5. Concentration-weighted trajectory (CWT) plots showing potential source regions of PM_2.5_ at the receptor site in Hangzhou (green dot) during the (a) pre-COVID, (b) Spring Festival, (c) COVID lockdown, and (d) post-COVID stages.


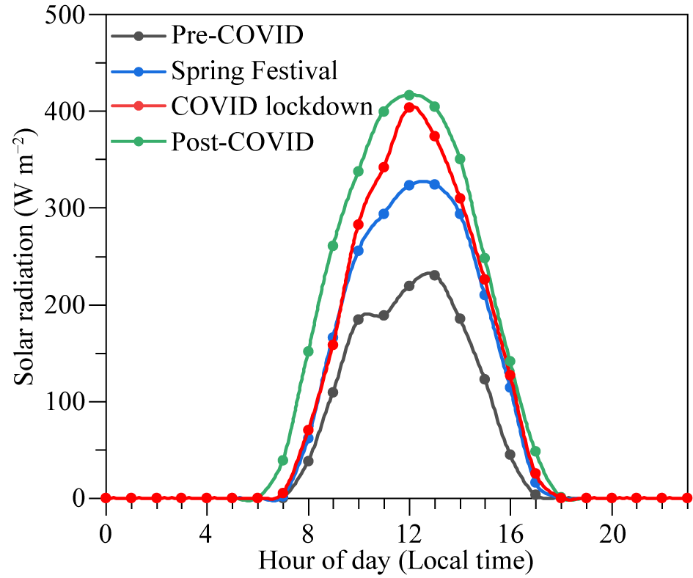


Figure S6. Diurnal variations of solar radiation during the pre-COVID, Spring Festival, COVID lockdown, and post-COVID stages.


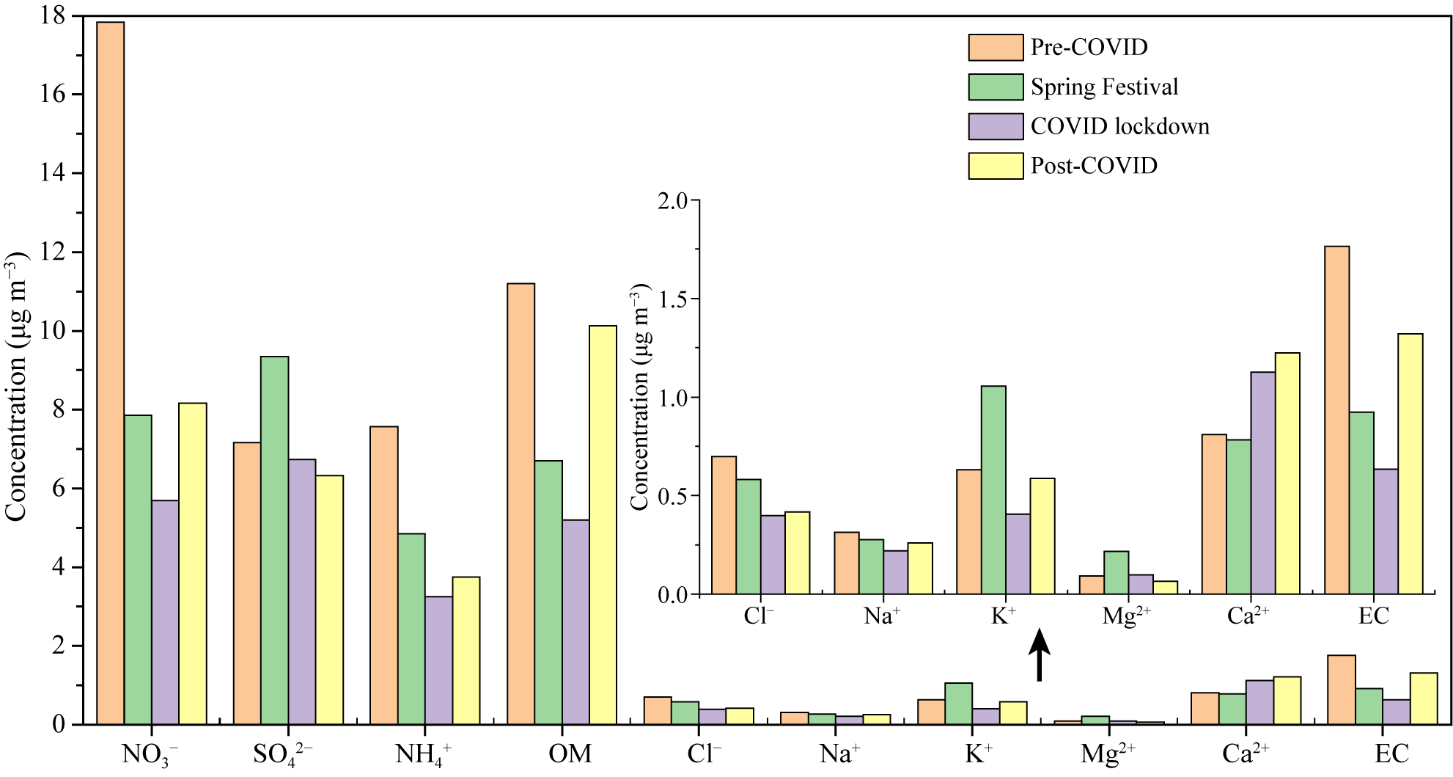


Figure S7. Comparisons of average concentrations of water-soluble ions, OM, and EC in PM_2.5_ among the pre-COVID, Spring Festival, COVID lockdown, and post-COVID stages.


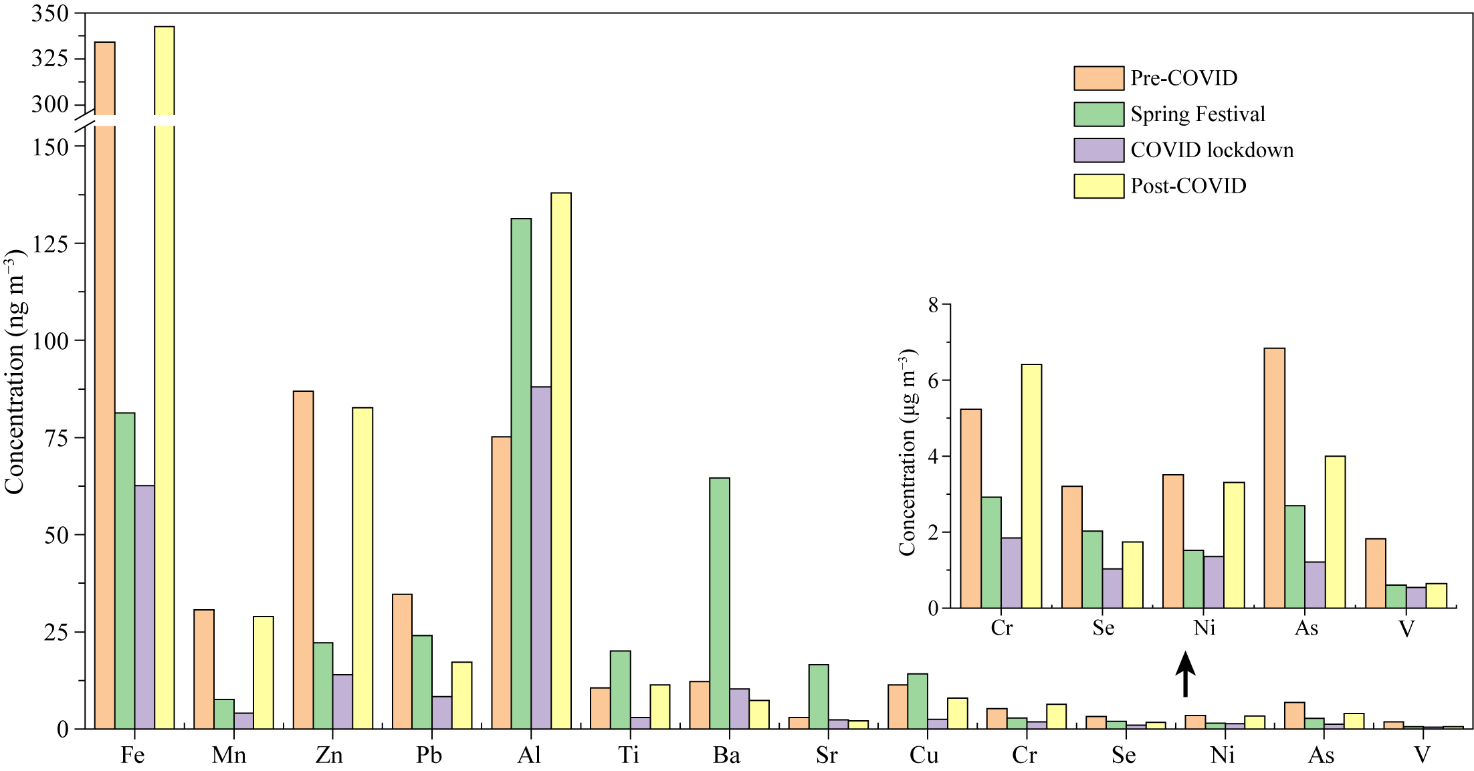


Figure S8. Comparisons of average concentrations of trace metals in PM_2.5_ among the pre-COVID, Spring Festival, COVID lockdown, and post-COVID stages.


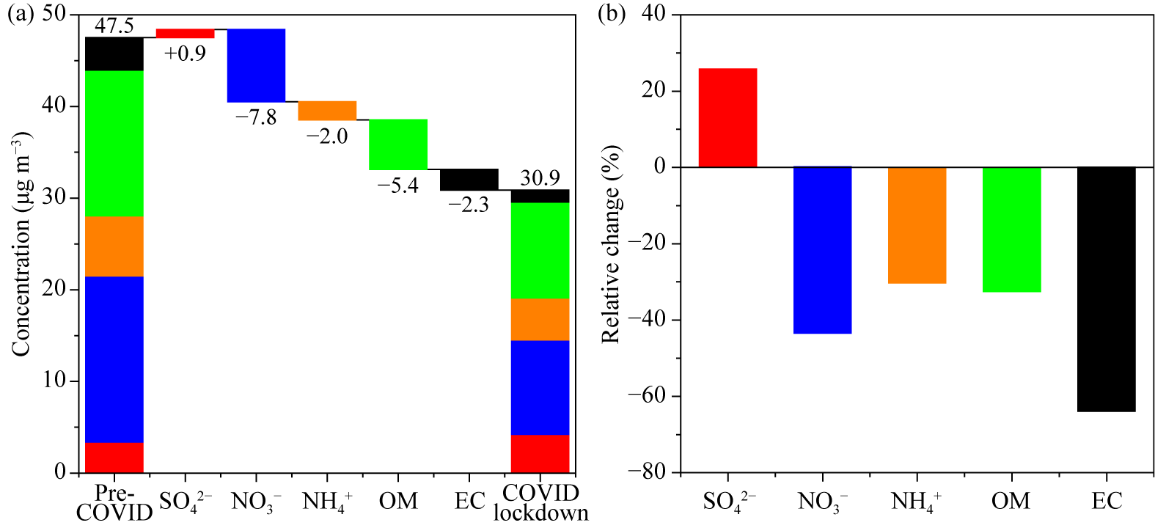


Figure S9. (a) Simulated average concentrations of major chemical species in PM_2.5_ during the pre-COVID and COVID lockdown stages and the concentration changes of each species between the two stages; (b) Relative changes of simulated major chemical species in PM_2.5_ between the two stages.

**Table S1.** Performance of the deweather model for the testing dataset.

| Species | n | FAC2 | MB | MGE | NMB | NMGE | RMSE | *r*^2^ | COE | IOA |
| --- | --- | --- | --- | --- | --- | --- | --- | --- | --- | --- |
| PM_2.5_ | 655 | 0.930 | 0.315 | 7.331 | 0.008 | 0.195 | 10.428 | 0.874 | 0.540 | 0.770 |
| PM_10_ | 655 | 0.934 | 0.353 | 9.965 | 0.007 | 0.189 | 14.231 | 0.860 | 0.547 | 0.774 |
| SO_2_ | 649 | 0.998 | 0.016 | 0.443 | 0.004 | 0.099 | 0.718 | 0.860 | 0.657 | 0.829 |
| CO | 649 | 1 | −1.182 | 70.640 | −0.001 | 0.065 | 97.546 | 0.873 | 0.558 | 0.779 |
| O_3_ | 629 | 0.870 | −0.736 | 8.157 | −0.015 | 0.169 | 11.820 | 0.872 | 0.649 | 0.825 |
| NO | 650 | 0.846 | −0.509 | 2.279 | −0.007 | 0.325 | 5.139 | 0.844 | 0.658 | 0.829 |
| NO_2_ | 650 | 0.954 | 0.134 | 6.121 | 0.004 | 0.171 | 9.333 | 0.862 | 0.650 | 0.825 |

Note: n—number of testing data, FAC2—fraction of predictions with a factor of two, MB—mean bias, MGE— mean gross error, NMB—normalized mean bias, NMGE—normalized mean gross, RMSE—root mean square error, *r*^2^—correlation coefficients, COE—Coefficient of Efficiency, IOA—Index of Agreement.

**Table S2.** Public health measures taken in China to prevent the spread of COVID-19 pandemic in January and February 2020. The main festivals in China are also given.

| Date | Location | Measures and main festivals in China |
| --- | --- | --- |
| 23 January 2020 | Wuhan and other 15 cities of Hubei Province | Lockdown on Wuhan and other cities in Hubei province; suspension of all public transport, control of movement***^a^*** |
|  | Zhejiang Province | First-level emergency response started on epidemic prevention and control |
| 24 January 2020 | China | Spring Festival’s Eve |
| 25 January 2020 | China | Spring Festival |
| 29 January 2020 | China | Fifth day of the first lunar month |
| 3 February 2020 | China | Tenth day of the first lunar month |
| 4 February 2020 | Hangzhou (Zhejiang) | Lockdown on Hangzhou; all the villages, communities, and government agencies in the city were under closed management; all public places were closed if they were unnecessary for inhabitants***^b^*** |
| 19 February 2020 | Hangzhou (Zhejiang) | All intercity highway checkpoints in Zhejiang province were cancelled; inner- and inter-city public transport was back to normal; parks, squares, and outdoor sports fields were opened***^c^*** |
|  |  | More than 80% of enterprises above designated size resumed production***^d^*** |

***^a^***en.wikipedia.org/wiki/2020_Hubei_lockdowns

***^b^***en.wikipedia.org/wiki/2019-20_coronavirus_pandemic_in_mainland_China

***^c^***http://www.hangzhou.gov.cn/art/2020/2/18/art_1228998465_41942552.html (accessed on 3 September 2020)

***^d^***http://huzcredit.huzhou.gov.cn/article/2020/02/article_bb4af4637008a41d017067c2bca502bf.htm (accessed on 3 September 2020)

**Table S3.** Summary of observed averages and standard deviations (Ave ± SD) of meteorological parameters and concentrations of particulate matter and gaseous pollutants during the pre-COVID, Spring Festival, COVID lockdown, and post-COVID stages.

|  | Pre-COVID | Spring Festival | COVID lockdown | Post-COVID |
| --- | --- | --- | --- | --- |
|  | Ave ± SD | Ave ± SD | Ave ± SD | Ave ± SD |
| Meteorological parameters | |  |  |  |
| Temperature (°C) | 7.5 ± 2.6 | 6.9 ± 1.2 | 8.4 ± 3.1 | 13 ± 3.9 |
| RH (%) | 82 ± 11 | 72 ± 18 | 76 ± 18 | 72 ± 14 |
| Wind speed (m s^−1^) | 2.0 ± 0.65 | 2.3 ± 0.58 | 2.0 ± 1.1 | 2.1 ± 0.69 |
| Particular matter and gaseous pollutants | | |  |  |
| PM_2.5_ (μg m^−3^) | 51.4 ± 30.2 | 36.2 ± 21.1 | 24.5 ± 9.2 | 34.1 ± 12.9 |
| PM_10_ (μg m^−3^) | 66.7 ± 37.8 | 41.3 ± 25.2 | 30.1 ± 8.8 | 57.8 ± 21.9 |
| SO_2_ (ppb) | 1.6 ± 0.68 | 1.3 ± 0.43 | 1.3 ± 0.36 | 1.7 ± 0.49 |
| CO (ppb) | 1040 ± 193 | 860 ± 112 | 769 ± 102 | 813 ± 109 |
| NO_x_ (ppb) | 37.5 ± 17.5 | 7.6 ± 2.6 | 6.1 ± 3.6 | 25.9 ± 9.2 |
| MDA8 O_3_ (ppb)*^a^* | 18.0 ± 10.7 | 39.5 ± 10.3 | 34.1 ± 12.3 | 37.3 ± 14.3 |

*^a^*MDA8 O_3_ represents the maximum daily 8-h average O_3_ concentration.

**Table S4.** Summary of deweathered average concentrations and standard deviations (Ave ± SD) of particulate matters and gaseous pollutants after decoupling the effects of meteorological conditions during the pre-COVID, Spring Festival, COVID lockdown, and post-COVID stages.

|  | Pre-COVID | Spring Festival | COVID lockdown | Post-COVID |
| --- | --- | --- | --- | --- |
|  | Ave ± SD | Ave ± SD | Ave ± SD | Ave ± SD |
| PM_2.5_ (μg m^−3^) | 52.3 ± 12.5 | 36.5 ± 8.7 | 26.3 ± 1.8 | 29.7 ± 4.3 |
| PM_10_ (μg m^−3^) | 68.4 ± 15.0 | 42.4 ± 8.9 | 34.2 ± 1.5 | 48.1 ± 6.9 |
| SO_2_ (ppb) | 1.7 ± 0.40 | 1.4 ± 0.07 | 1.4 ± 0.06 | 1.7 ± 0.16 |
| CO (ppb) | 1023 ± 106 | 865 ± 55 | 782 ± 17 | 812 ± 31 |
| NO_x_ (ppb) | 35.9 ± 12.8 | 9.4 ± 1.0 | 8.1 ± 1.7 | 25.3 ± 5.4 |
| MDA8 O_3_ (ppb)*^a^* | 14.4 ± 3.9 | 30.7 ± 0.8 | 27.9 ± 2.4 | 24.3 ± 2.1 |

*^a^*MDA8 O_3_ represents the maximum daily 8-h average O_3_ concentration.

**Table S5.** Summary of average concentrations and standard deviations (Ave ± SD) of major chemical components in PM_2.5_ during the pre-COVID, Spring Festival, COVID lockdown, and post-COVID stages.

| (μg m^−3^) | Pre-COVID | Spring Festival | COVID lockdown | Post-COVID |
| --- | --- | --- | --- | --- |
|  | Ave ± SD | Ave ± SD | Ave ± SD | Ave ± SD |
| OM | 11.2 ± 4.8 | 6.9 ± 3.8 | 5.2 ± 2.1 | 10.1 ± 3.8 |
| EC | 1.8 ± 0.74 | 0.91 ± 0.49 | 0.64 ± 0.28 | 1.3 ± 0.56 |
| NO_3_^−^ | 17.8 ± 12.4 | 7.8 ± 5.8 | 5.7 ± 2.9 | 8.2 ± 5.5 |
| SO_4_^2−^ | 7.1 ± 3.3 | 9.3 ± 5.4 | 6.7 ± 2.1 | 6.2 ± 2.0 |
| Cl^−^ | 0.70 ± 0.50 | 0.60 ± 0.81 | 0.40 ± 0.26 | 0.42 ± 0.29 |
| NH_4_^+^ | 7.6 ± 4.8 | 5.1 ± 3.6 | 3.2 ± 1.8 | 3.8 ± 2.2 |
| K^+^ | 0.63 ± 0.28 | 1.1 ± 0.73 | 0.41 ± 0.18 | 0.59 ± 0.18 |
| Na^+^ | 0.31 ± 0.18 | 0.27 ± 0.19 | 0.22 ± 0.12 | 0.26 ± 0.13 |
| Mg^2+^ | 0.09 ± 0.08 | 0.24 ± 0.17 | 0.08 ± 0.06 | 0.07 ± 0.06 |
| Ca^2+^ | 0.81 ± 0.30 | 0.75 ± 0.40 | 1.1 ± 0.28 | 1.2 ± 0.24 |
| Trace metals | 0.62 ± 0.29 | 0.38±0.23 | 0.20 ± 0.06 | 0.66 ± 0.21 |

**Table S6.** Summary of average concentrations and standard deviations (Ave ± SD) of major metals in PM_2.5_ during the pre-COVID, Spring Festival, COVID lockdown, and post-COVID stages.

| (ng m^−3^) | Pre-COVID | Spring Festival | COVID lockdown | Post-COVID |
| --- | --- | --- | --- | --- |
|  | Ave ± SD | Ave ± SD | Ave ± SD | Ave ± SD |
| Fe | 334±189 | 81±40 | 63±30 | 343±122 |
| Mn | 31±19 | 7.6±5.4 | 4.1±2.5 | 29±14 |
| Zn | 87±52 | 22±14 | 14±6.4 | 83±38 |
| Pb | 35±20 | 24±24 | 8.3±3.8 | 17±6.2 |
| Al | 75±43 | 131±55 | 88±18 | 138±63 |
| Ti | 11±9.9 | 20±20 | 3.0±3.3 | 11±6.5 |
| Ba | 12±10 | 65±62 | 10±9.5 | 7.3±1.5 |
| Sr | 3.0±2.2 | 17±17 | 2.4±3.2 | 2.1±0.8 |
| Cu | 11±6.9 | 14±14 | 2.5±3.0 | 8.0±3.1 |
| Cr | 5.2±4.0 | 2.9±1.2 | 1.8±0.5 | 6.4±2.8 |
| Se | 3.2±2.0 | 2.0±1.5 | 1.0±0.8 | 1.8±1.1 |
| Ni | 3.5±1.6 | 1.5±0.7 | 1.4±0.5 | 3.3±1.4 |
| As | 6.8±4.6 | 2.7±2.2 | 1.2±0.8 | 4.0±1.9 |
| V | 1.8±3.9 | 0.6±0.9 | 0.5±0.4 | 0.6±0.5 |

**References:**

Fleming, Z. L., Monks, P. S., & Manning, A. J. (2012). Review: Untangling the influence of air-mass history in interpreting observed atmospheric composition. *Atmospheric Research*, *104-105*, 1-39. http://doi.org/10.1016/j.atmosres.2011.09.009

Grange, S. K., Carslaw, D. C., Lewis, A. C., Boleti, E., & Hueglin, C. (2018). Random forest meteorological normalisation models for Swiss PM_10_ trend analysis. *Atmospheric Chemistry and Physics*, *18*(9), 6223-6239. http://doi.org/10.5194/acp-18-6223-2018

Grell, G. A., Peckham, S. E., Schmitz, R., McKeen, S. A., Frost, G., Skamarock, W. C., & Eder, B. (2005). Fully coupled “online” chemistry within the WRF model. *Atmospheric Environment*, *39*(37), 6957-6975. http://doi.org/10.1016/j.atmosenv.2005.04.027

Huang, X., Ding, A., Gao, J., Zheng, B., Zhou, D., Qi, X., et al. (2020). Enhanced secondary pollution offset reduction of primary emissions during COVID-19 lockdown in China. *National Science Review*, nwaa137. http://doi.org/10.1093/nsr/nwaa137

Li, M., Zhang, Q., Streets, D. G., He, K. B., Cheng, Y. F., Emmons, L. K., et al. (2014). Mapping Asian anthropogenic emissions of non-methane volatile organic compounds to multiple chemical mechanisms. *Atmospheric Chemistry and Physics*, *14*(11), 5617-5638. http://doi.org/10.5194/acp-14-5617-2014

Petit, J. E., Favez, O., Albinet, A., & Canonaco, F. (2017). A user-friendly tool for comprehensive evaluation of the geographical origins of atmospheric pollution: Wind and trajectory analyses. *Environmental Modelling & Software*, *88*, 183-187. http://doi.org/10.1016/j.envsoft.2016.11.022

Polissar, A. V., Hopke, P. K., & Poirot, R. L. (2001). Atmospheric aerosol over Vermont: Chemical composition and sources. *Environmental Science & Technology*, *35*(23), 4604-4621. http://doi.org/10.1021/es0105865

Stein, A. F., Draxler, R. R., Rolph, G. D., Stunder, B. J. B., Cohen, M. D., & Ngan, F. (2016). NOAA’s HYSPLIT Atmospheric Transport and Dispersion Modeling System. *Bulletin of the American Meteorological Society*, *96*(12), 2059-2077. http://doi.org/10.1175/bams-d-14-00110.1

Vu, T. V., Shi, Z., Cheng, J., Zhang, Q., He, K., Wang, S., & Harrison, R. M. (2019). Assessing the impact of clean air action on air quality trends in Beijing using a machine learning technique. *Atmospheric Chemistry and Physics*, *19*(17), 11303-11314. http://doi.org/10.5194/acp-19-11303-2019

Wang, Y. Q., Zhang, X. Y., & Draxler, R. R. (2009). TrajStat: GIS-based software that uses various trajectory statistical analysis methods to identify potential sources from long-term air pollution measurement data. *Environmental Modelling & Software*, *24*(8), 938-939. http://doi.org/10.1016/j.envsoft.2009.01.004
